# Low rate of high-level athletes maintained a return to pre-injury sports two years after arthroscopic treatment for femoroacetabular impingement syndrome

**DOI:** 10.1186/s40634-020-00263-5

**Published:** 2020-06-25

**Authors:** Josefin Abrahamson, Ida Lindman, Mikael Sansone, Axel Öhlin, Pall Jonasson, Jón Karlsson, Adad Baranto

**Affiliations:** Department of Occupational Orthopedics and Research, Institute of Clinical Sciences at Sahlgrenska Academy University of Gothenburg and Sahlgrenska University Hospital Hospital/ Mölndal Hospital, R-house, Floor 7, Mölndal, SE-431 80 Gothenburg, Sweden

**Keywords:** Hip arthroscopy, Femoroacetabular impingement, Return to sport, Elite athletes

## Abstract

**Purpose:**

The aim was to investigate the rate of athletes still active at their pre-injury sports level two years after arthroscopic treatment for femoroacetabular impingement syndrome (FAIS), and examine this between different sports and gender, and its correlation to patient-reported outcome measures (PROMs).

**Method:**

High-level athletes planned for arthroscopic treatment for FAIS were included prospectively in a Swedish hip arthroscopy registry between 2011 and 2017, and 717 met the inclusion criteria. Self-reported sporting activity was recorded preoperatively. The subjects answered PROMs, including the HSAS, iHOT-12 and HAGOS pre- and postoperatively.

**Results:**

A total of 551 athletes (median age 26, interquartile range 20–34 years; 23% women) had completed follow-up PROMs, at mean 23.4 ± 7.2 months postoperatively. In total, 135 (24.5%) were active at their pre-injury level of sports at follow-up (RTS^pre^). Athletes ≤30 years at time of surgery (*n* = 366; median age 22 years) had higher rate of RTS^pre^ (31.4%) compared with athletes > 30 years (*n* = 185; median age 40 years) (10.8%; *p* < 0.001). All athletes had improvements in iHOT-12 and HAGOS, two years postoperatively (p < 0.001), while RTS^pre^ athletes reported significantly better PROMs, pre- and postoperatively, and had greater improvements two years postoperatively, compared with athletes not active at pre-injury level.

**Conclusion:**

Only 25% of all high-level athletes and 31% of athletes ≤30 years were still active at their pre-injury sports level two years after arthroscopic treatment for FAIS. Athletes still active had significantly and clinically greater improvement regarding hip symptoms, function and quality of life, as compared with athletes not active at pre-injury level, two years postoperatively.

## Introduction

Femoroacetabular impingement syndrome (FAIS) is a clinical disorder of the hip joint that has been shown to be common cause of hip pain among athletes [1]. FAIS comprises abnormal morphology in the hip joint, either at the femoral head-neck junction (cam) and/or at the acetabulum (pincer), combined with hip pain and clinical findings of reduced hip joint flexion and/or internal rotation and/or positive impingement tests [2]. At present, it is thought that cam morphology development is greatly affected by participation in high-impact sports during skeletal maturation [3–5]. Sports with repetitive axial loads, hip flexion with/without internal rotation, such as ice-hockey and soccer, appear to put the hips at high risk [6]. Moreover, there is strong evidence that FAIS is a major risk factor for hip osteoarthritis (OA) [7, 8].

Arthroscopic surgery is an increasingly common treatment in patients with FAIS and has emerged as an established treatment in these patients during the last 10–15 years [9–11]. Patient-reported outcome measurements (PROMs) of hip function, pain, sports function, quality of life (QoL) and activity of daily living (ADL) after hip arthroscopy appear to be generally good, in both the short and long term [11–13]. The main goal of most athletes undergoing surgical treatment for FAIS is to return to sport (RTS) at their pre-injury level [14]. Systematic reviews have reported rates of RTS after surgical treatment (mainly arthroscopy) of between 87 and 92%, with return to the same level ranging between 74 and 88% [9, 15, 16]. However, both the criteria for RTS and the methods used to investigate it have been reported with a wide variety and often with unclear definitions, which likely affects the outcome for RTS. In a recent consensus statement, Ardern et al. recommended a three grade continuum in the assessment of RTS, including return to participation (lower level), return to sport (RTS, but not at pre-injury/desired performance level) and return to performance (fully returned to pre-injury/higher level) [14]. Furthermore, many studies define that RTS has been reached when the athlete has been able to participate in one game/competition or one season (either at any level or at pre-injury level), at one single occasion during the follow-up period [17–19]. This means that the athlete’s ability to keep continue be at their pre-injury level, at longer terms, may be missed out.

The aim of this study was to investigate the rate of high-level athletes who are at their pre-injury level of sports two years after arthroscopic treatment for FAIS. Moreover, the aim was also to investigate return rate between different sports and genders, and analyze its correlation to patient-reported outcome measures (PROMs).

## Material and methods

### Study design and setting

Ethical approval was granted by the Regional Ethical Review Board in Gothenburg, Sahlgrenska Academy, Gothenburg University, Sweden (registration number 071–12). The study was conducted in agreement with the Helsinki Declaration. This study uses a longitudinal analysis to investigate the rate of athletes who were still active at their pre-injury sport level (defined in this study as RTS^pre^) two years postoperatively. Subjects were identified in a hip outcome registry including all hip arthroscopies performed in an out-patient setting at two orthopedic centers in Gothenburg, Sweden. Participants were prospectively recruited from December 2011 to March 2017.

### Participants

**Fig. 1:**
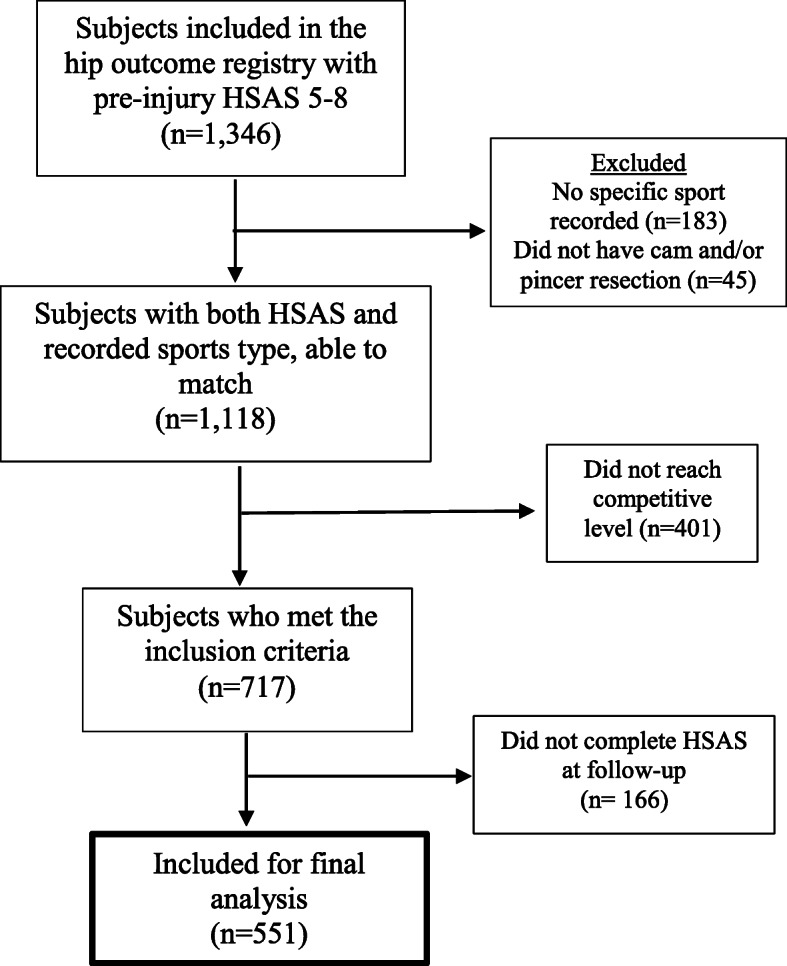
Flow-chart of participants

All high-level athletes who had arthroscopic surgery for FAIS with a recorded type of sports activity were included in the study (Fig. 1). The inclusion criteria were participation in high-level sports before the onset of initial hip pain (i.e. pre-injury) and undergoing arthroscopic treatment for FAIS consisting of cam and/or pincer resection. Severe dysplasia (lateral center edge angle ≤ 20°) or advanced OA (joint space < 2 mm) were contraindications for surgery. “High-level” was defined as being at a competitive level with regard to the Hip Sports Activity Scale Outcome Score (HSAS) (See additional file 1, Table A1) [20]. To identify these athletes, their self-reported sports activity was matched to their pre-injury HSAS score and categorized as either 1) Competitive elite (national or international elite level) or 2) Competitive sub-elite (lower divisions) (Table 1). For example, a golf player who scored HSAS 6 was categorized as competitive elite level, while a soccer player who scored HSAS 6 did only reach recreational level and was therefore excluded. Only athletes who, pre-injury, reached competitive elite or sub-elite level (HSAS 5–8 depending on sport type) and had hip arthroscopy consisting of cam and/or pincer resection were included. The exclusion criteria were athletes who did not reach competitive level (HSAS 0–6, depending on sport type, Table 1) or had missing data on sports activity or HSAS pre-injury score.

**Table 1:** Included and excluded athletes relative to their sport type and pre-injury HSAS score

Sports type	Pre-injury HSAS	Included/excluded
Soccer, ice hockey, field hockey, American football/rugby, martial arts, tennis, track and field, indoor sports, beach volleyball, lacrosse, baseball/softball	**8**	Included as elite level athletes
	**7**	Included as sub-elite level athletes
	**0–6**	Excluded due to recreational level
Downhill skiing, snowboarding, figure skating, skates, dancing	**7–8**	Included as elite level athletes
	**6**	Included as sub-elite level athletes
	**0–5**	Excluded due to recreational level
Golf, bicycle racing, mountain biking, swimming, rowing, cross-country skiing/biathlon, horseback riding, cricket	**6–8**	Included as elite level athletes
	**5**	Included as sub-elite level athletes
All	**0–4**	Excluded due to recreational level

Patient history, clinical and radiological findings consistent with the FAIS, set the diagnosis of FAIS [2]. The surgical technique has previously been described [21]. In short, the patient was in a supine position and mid-anterior and antero-lateral portals were used. To view the central compartment, axial traction was achieved and, to access the peripheral compartment, a ligament-sparing capsulotomy was performed. A cam resection was made, between lateral, medial/caudal and posterior, with an intraoperative dynamic assessment of impingement, to prevent residual impingement. In the event of posterior impingement (pistol grip deformity), a posterior and cranial resection was also made to the lateral retinacular fold. Care was always taken to preserve the lateral retinacular vessels. Pincer deformity was removed with an “over-the-top” technique, where the acetabular edge was resected with the labrum in situ*.* In cases of larger resections or labral detachment, the labrum was re-attached. To avoid heterotopic ossification, the patients took non-steroidal anti-inflammatory drugs (NSAID) for one month. Physical therapy started directly postoperatively and the patients were allowed free ROM, full weight-bearing and were recommended to use crutches for four weeks after surgery in outdoor and longer ambulation. The rehabilitation protocol included exercises for ROM, strengthening, endurance, balance and coordination with gradually increased intensity, as tolerated by the patient.

### Data collection

Preoperatively, all athletes completed a set of web-based, self-administered patient-reported outcome measures (PROM) including the HSAS [20] before symptom onset (pre-injury), the International Hip Outcome Tool (iHOT-12) [22] and the Copenhagen Hip and Groin Outcome Score (HAGOS) [23]. Age, duration of symptoms, sports activity and procedures performed (cam and/or pincer resection), were recorded at time of surgery. The athletes did complete the same set of web-based, self-administered PROMs two years postoperatively with the exception of the HSAS, that was recorded *at present* and not pre-injury. The iHOT-12, HAGOS and HSAS have previously been adapted and validated to Swedish [24–26].

### Statistical analysis

Data were analyzed using the SPSS (version 25.0, Armonk, NY: IBM Corp) software package. Normal distribution was tested with the Kolmogorov-Smirnov test and, due to skewed data distributions, descriptive results were presented as the median (interquartile range Q_25_-Q_75_, IQR), unless otherwise stated. The Mann-Whitney *U* test was used for comparisons. The chi^2^ test was used for differences between categorical data and Wilcoxon’s signed rank test was used for repeated measures between preoperative and the postoperative follow-up. The significance level was set at *P* < 0.05. To assess RTS^pre^, the athletes HSAS score pre-injury was compared with their HSAS score postoperatively. If they obtained the same or a higher HSAS score postoperatively, as compared to their pre-injury HSAS score, they were included in the RTS^pre^ group. If they scored lower HSAS postoperatively (as compared to their pre-injury HSAS), they were included in the no-RTS^pre^ group. Only data from athletes with both pre- and postoperative outcomes scores were used. A subgroup analysis was made, comparing athletes 30 years or younger (≤30 years) with athletes older than 30 years (> 30 years). To asses clinically relevant change, the minimal important change (MIC) was used for HAGOS six subscales and iHOT-12. MIC has previously been described to be for HAGOS symptoms 9.3, pain 9.7, ADL 11.8, sports 10.8, physical activities 13.1, and QoL 8.8 [25] and with 9.0 for iHOT-12 [24], and was used in this study. In addition, the patient acceptable symptomatic state (PASS) was also used for iHOT-12, and has been described as 63.0 [27].

## Results

Five-hundred and fifty-one of 717 (response rate 76.8%) had postoperative follow-up data, with a mean follow-up of 23.4 (±7.2) months. Isolated cam resection was seen in 183 athletes (33.2%); six athletes (1.1%) had isolated pincer resection; and 362 (65.7%) had a combination of cam and pincer resections. Table 2 shows demographics and comparisons of preoperative data between male and female patients, and between the two age groups (≤30 years; *n* = 366, 66.4% and > 30 years; *n* = 185). There was a significant difference between responders and non-responders in terms of gender (male: 74% vs. 26%; women: 86% vs. 14% [*p* = 0.002]), duration of symptoms (24, IQR 12–60 vs. 24, IQR 12–36 months [*p* = 0.02]) and preoperative score of HAGOS subscale symptoms (43, IQR 29–61 vs. 50, IQR 36–61 [p = 0.02]). No differences were seen for age, level of sport or preoperative scores on the iHOT-12 or the other five HAGOS subscales.

**Table 2:** Patient demographics and preoperative data between gender and the two ages groups, respectively

	Male (***n*** = 425)	Female (***n*** = 126)	***P*** value	≤30 years (***n*** = 366)	> 30 years (***n*** = 185)	***P*** value
Age, years	26 (21–34)	24 (19–34)	0.1^1^	22 (19–25)	40 (34–46)	**< 0.001**^**a**^
Females, n (%)	–	–	–	101 (20.4)	45 (20.2)	0.9^2^
Level of sport, n (%)						
Elite level	235 (55.3)	70 (55.6)	1.0^2^	297 (60.1)	112 (50.5)	**0.02**^**b**^
Sub-elite level	189 (44.7)	56 (44.4)		197 (39.9)	110 (49.5)	
Duration of symptoms, months	24 (12–48)	33 (18–60)	**0.07**^**1**^	24 (12–48)	36 (24–48)	**< 0.001**^**a**^

Values in median (IQR, Q_25_-Q_75_), unless specified. ^a^Mann-Whitney *U* test. ^b^Chi^2^-test. **Bold**, indicates a significant difference

Nearly 25% (135 of 551) of the athletes were at the same or a higher level of sports (RTS^pre^) at follow-up, 72% were active in sport at lower levels and 3% reported no sporting activity. Table 3 shows the summarized RTS^pre^ for all sports and ages, as well as stratified into age groups and for the three most common sport types in this cohort (soccer, ice hockey and horseback riding). RTS^pre^ was highest in athletes ≤30 years (31.4%, *p* < 0.001). Horseback riders had the highest RTS^pre^ (34.5%), with an additional different pattern between the age groups. The duration of symptoms was significantly shorter in RTS^pre^ (median 24, IQR 12–48 months) compared with no-RTS^pre^ (median 24, IQR 16–60 months; p < 0.001). This was not shown when stratified into age groups. There were no differences in RTS^pre^ between gender (males 26.1% vs females 19.0%) or elite and sub-elite athletes (25.9% vs 22.9%).

**Table 3:** RTS^pre^ and no-RTS^pre^ for the entire group, the most common sports and by age groups

	RTS^pre^	no-RTS^pre^
All sports (*n* = 551)	135 (24.5)	416 (75.5)
≤ 30 years (n = 366)	115 (31.4)	251 (68.6)
> 30 years (n = 185)	20 (10.8)	165 (89.2)
Soccer (*n* = 230)	62 (27.0)	168 (73.0)
≤ 30 years (*n* = 176)	61 (34.7)	115 (65.3)
> 30 years (*n* = 54)	1 (1.9)	53 (98.1)
Ice hockey (*n* = 82)	20 (24.4)	62 (75.6)
≤ 30 years (*n* = 57)	18 (31.6)	39 (68.4)
> 30 years (*n* = 25)	2 (8.0)	23 (92.0)
Horseback riding (*n* = 29)	10 (34.5)	19 (65.5)
≤ 30 years (*n* = 13)	5 (38.5)	8 (61.5)
> 30 years (*n* = 16)	5 (31.3)	11 (68.6)

Values in number (%). RTS^pre^, active at pre-injury level, no-RTS^pre^, not active at pre-injury level

### PROMs versus RTS^pre^

**Fig. 2:**
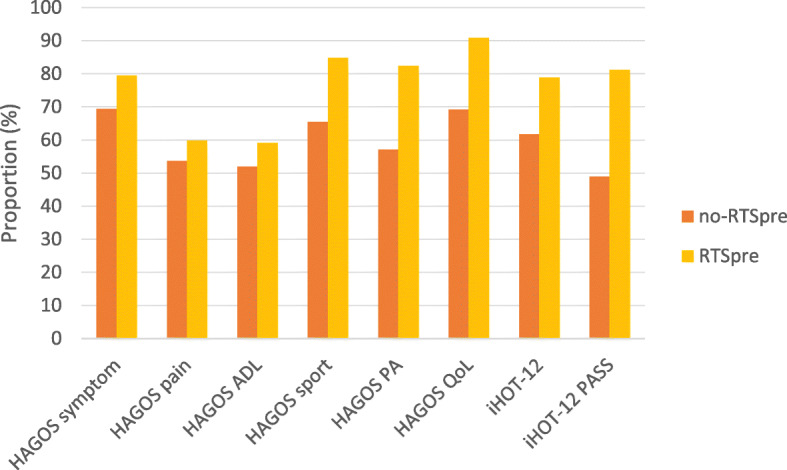
Proportion (%) of RTS^pre^ and no-RTS^pre^ athletes reaching MIC in HAGOS six subscales and iHOT-12, and PASS in iHOT-12, two years postoperatively. RTS^pre^, active at pre-injury level; no-RTS^pre^, not active at pre-injury level; ADL, Activity of Daily Living; PA, Physical Activities; QoL, Quality of Life; MIC, minimal important change; PASS, patient acceptable symptomatic state

Table 4 shows comparisons between RTS^pre^ and no-RTS^pre^ for iHOT-12 and HAGOS six subscales pre- and postoperative, and their change from pre- to the postoperative follow-up, respectively. All the athletes reported a significant improvement in both the iHOT-12 and all six HAGOS subscales, between pre- and two years postoperatively (*p* < 0.001). This improvement was significantly greater for RTS^pre^ in iHOT-12 and all HAGOS subscales, except for ADL, as compared with no-RTS^pre^ athletes (Table 4). The same was found when stratified into age groups. A higher proportion of RTS^pre^ reached MIC and PASS in all PROMs as compared with no-RTS^pre^ (Fig. 2).

**Table 4:** iHOT-12 and HAGOS subscales for RTS^pre^ compared with no-RTS^pre^ preoperatively and two years postoperatively^a^

	Preoperative		Postoperative		Change (Δ)	
	RTS^pre^	no-RTS^pre^	RTS^pre^	no-RTS^pre^	RTS^pre^	no-RTS^pre^
iHOT-12	46 (33–65)	41 (29–52)^**^	50 (30–80)	80 (60–95)^*^	29 (11–47)	19 (1–42)^*^
HAGOS						
Symptom	46 (29–64)	43 (29–57)	82 (71–93)	68 (50–82)^*^	28 (11–50)	21 (4–39)^*^
Pain	73 (53–88)	55 (45–78)^*^	90 (83–100)	78 (55–93)^*^	18 (0–32)	13 (−5–30)^***^
ADL	75 (55–95)	60 (42–80)^*^	100 (90–100)	85 (60–100)^*^	15 (0–40)	15 (0–39)
Sport	35 (22–56)	31 (19–50)	88 (69–100)	67 (41–88)^*^	38 (22–66)	28 (3–50)^*^
PA	25 (13–50)	13 (0–38)^**^	88 (75–100)	50 (13–88)^*^	51 (25–75)	25 (0–63)^*^
QoL	30 (15–48)	25 (15–40)^***^	80 (60–95)	50 (30–80)^*^	45 (22–65)	25 (5–50)^*^

Values in median (IQR, Q_25_-Q_75_). RTS^pre^, active at pre-injury level two years postoperatively, no-RTS^pre^, not active at pre-injury level two years postoperatively. *ADL* Activity of Daily Living, *PA* Physical Activities, *QoL* Quality of Life. ^a^Mann Whitney *U* test used to compare RTS^pre^ and no-RTS^pre^: preoperatively, postoperatively and change from pre- to the postoperative follow-up, respectively. Significant difference between RTS^pre^ and no-RTS^pre^ indicated by ^*^p < 0.001; ^**^p = 0.003; ^***^p < 0.03

## Discussion

The most interesting findings in this study were that only 25% (135 of 551) of all high-level athletes, and 31.4% (115 of 366) of those athletes 30 years or younger, were active at their pre-injury level two years after arthroscopic treatment for FAIS. High-level athletes older than 30 years, had significantly lower rate of RTS^pre^ (10.8%). Furthermore, self-reported hip function was improved in all athletes in all PROMs, from preoperative to the postoperative follow-up. However, RTS^pre^ athletes reported, significant and clinical, greater improvements in nearly every PROMs from preoperative to the postoperative follow-up, as compared with no-RTS^pre^ athletes.

Only 135 of 551 (25%) high-level athletes, and 115 of 366 (31.4%) athletes ≤30 years, who underwent arthroscopic treatment for FAIS were active at their pre-injury level or higher two years postoperatively. These results are comparable with recent studies by Wörner et al., Tjissen et al. and Ishöi et al. [28–30]. Wörner et al. and Tjissen et al. investigated the return rate in 127 and 37 athletes after arthroscopic treatment for FAIS, respectively [29, 30]. The studies showed that 84–89% had returned to some kind of sport, yet only 19–21% were classified as RTS^pre^. Moreover, Ishöi et al. investigated 189 athletes at a mean of 33 months after hip arthroscopy [28]. Fifty-seven percent returned to the same sport at pre-injury level, although only 30% of these (17% of the whole study population) were estimated to have returned to their optimal performance. In contrast, compared with earlier reports from systematic reviews, these results are low [9, 15, 16]. The main cause of the disparity in relation to the results in this study is probably the wide variation in the definition of *return to sport* that is used, how it is measured and the time point of follow-up. Many studies have follow-up periods that span several years [16]. However, these studies often assess a successful RTS or RTS^pre^ by if the athlete reach the RTS goal at some time point during the follow-up period. This may only indicate the athletes’ ability to RTS at short term, and not their capacity of maintain RTS at mid- or long term. Menge et al. and Frangiamore et al. defined successful return to sport as if the athlete was playing in a single season game after the hip arthroscopy for FAIS, giving RTS rates of 87% and 95%, respectively [18, 19]. This definition reports that the athletes are able to play one game, yet their ability of prolonged performance are unknown. These two studies did also report career length after RTS with mean 3.6 ± 2.9 and 3.2 ± 2.1 seasons played, respectively [18, 19], which gives a certain indication of their persistent ability to play. Furthermore, Christian et al. also reported high rates of RTS (81.0–95.9%) in a cohort of 131 professional athletes undergone hip arthroscopy (type of hip injuries were not specified) [17]. This study had the same definition of RTS as the two studies above and reported fairly few seasons played postoperatively, median ranged between 0.9–2.7. The present study did not investigate if the athletes did return to their pre-injury level at any earlier point in time, as it aimed to investigate the rate of athletes who were active at their pre-injury level at two years postoperatively. This is probably the main cause of the discrepancy between study results.

Horseback riders differed from participants in the other two sports. Although that they were slightly older (majority > 30 years), they still had the highest rate of RTS^pre^. One explanation for the high rate of RTS^pre^ might be that, when the bony source of the impingement is removed, horseback riding may be less provocative for the hips compared with other sports. It is also worth speculating whether cam and/or pincer morphologies have the same speed of progression to create intra-articular damage, hip symptoms and dysfunction, that may lead to arthroscopic treatment in different sports, as the horseback riders were older. Sports that are not specific high-impact sports and not include elements of pivoting and cutting, such as riding, may have different development pattern, from having only the radiological features of cam/pincer morphology to have all the diagnostic criteria of FAIS, and may also have lesser postoperative problems. Furthermore, a reason why riders were shown to have higher rates of RTS^pre^, even in the older age group, may be the age of peak competitive performance that appears to be at 34–36 years in riders [31, 32], and might motivate this population to return to a higher degree.

All the athletes in this study had improved self-reported hip function, shown in all PROMs, from preoperative to the two years postoperative follow-up. This is in line with earlier studies [10, 33], and indicate that the overall quality of surgery and rehabilitation was good. However, in this study there was a significant difference in nearly every PROMs between RTS^pre^ and no-RTS^pre^, both pre- and postoperatively in favor for RTS^pre^. These athletes (RTS^pre^) were also shown greater improvement from pre- to the postoperative follow-up, as well as with higher proportion that reached MIC and PASS in all PROMs. One explanation for differences in PROMs could be that RTS^pre^ athletes might have good hip function and fewer symptoms and pain, and therefore be able to attain their pre-injury level of sports. Furthermore, it is also worth speculating about whether the lower PROMs reported by no-RTS^pre^ is due to their hip problems limiting their opportunities for sports performance or the desired level of sports. The duration of symptoms did differ between RTS^pre^ (24, IQR 12–48 months) and no-RTS^pre^ (24, IQR 16–60 months). An association between symptom duration and RTS or career length has previously been reported [34–36], even though the cause of this association is not known. This may be due to that they may have been unable to train and compete for years, limiting the opportunity to return to elite level, or other life choices affecting their level of sports during such a long time period.

### Strengths and limitations

One of the main strengths of the present study is the large number of participants. Another strength is the prospectively collected data that provided an opportunity to compare preoperative data with those from postoperative. This might also reduce the risk of recall bias, even though this exists, as at the time of surgery, the athletes estimate their HSAS as it was pre-injury. To evaluate RTS^pre^, the HSAS was used; it has been translated and validated in Swedish [26]. The HSAS has shown good validity [20] and been used in previous studies of RTS [35, 37]. However, this outcome tool might be fairly difficult to use if, for example, the respondent performs a type of sport the HSAS does not mention and this can be regarded as a limitation of this study. The HSAS does not specify the dose of loading or other reasons why the level of activity is lower. The present study did not exclude athletes by their intention to return or not to return to their pre-injury level. This may be one of the main factors in explaining why athletes do or do not return to sport and might therefore influence the outcomes in the present study. Future studies should use a strict definition of RTS, including the question of the athlete’s intention to return to their pre-injury sport level. Cartilage status was not included in this study and this can be regarded as a limitation. There are, however, contraindicatory results when it comes to whether or not cartilage damage influences RTS/career length after arthroscopic treatment for FAIS [15, 34, 36]. The hip registry used in the present study only includes patients from two single high-volume hip arthroscopy centers in Gothenburg, which may reduce the generalizability to other regions. On the other hand, having a few surgeons and similar surgical approaches allows for generalizability in the follow-up analysis of RTS and PROMs and could be regarded as a strength.

## Conclusions

Two years after the arthroscopic treatment for FAIS, 25% of all high-level athletes, and 31% in athletes 30 years or younger, were still active at their pre-injury sports level. Those still active at pre-injury level, were shown to have higher PROMs pre- and postoperatively, as well as greater clinically relevant improvements from pre- to the postoperative follow-up.

## Supplementary information

**Additional file 1.**

## Abbreviations

FAIS: Femoroacetabular impingement syndrome
RTS^pre^: Still active at pre-injury sports level
HSAS: Hip Sports Activity Scale Outcome Score
iHOT12: International Hip Outcome Tool
HAGOS: Copenhagen Hip and Groin Outcome Score
ROM: Range of motion

## References

[1] Larson CM, Sikka RS, Sardelli MC, Byrd JT, Kelly BT, Jain RK, Giveans MR (2013). Increasing alpha angle is predictive of athletic-related “hip” and “groin” pain in collegiate National Football League prospects. Arthroscopy.

[2] Griffin D, Dickenson E, O'Donnell J, Awan T, Beck M, Clohisy J, Dijkstra H, Falvey E, Gimpel M, Hinman R (2016). The Warwick agreement on femoroacetabular impingement syndrome (FAI syndrome): an international consensus statement. Br J Sports Med.

[3] Agricola R, Heijboer MP, Ginai AZ, Roels P, Zadpoor AA, Verhaar JA, Weinans H, Waarsing JH (2014). A cam deformity is gradually acquired during skeletal maturation in adolescent and young male soccer players: a prospective study with minimum 2-year follow-up. Am J Sports Med.

[4] Jónasson PS, Ekström L, Hansson H-A, Sansone M, Karlsson J, Swärd L, Baranto A (2015) Cyclical loading causes injury in and around the porcine proximal femoral physeal plate: proposed cause of the development of cam deformity in young athletes. J Exp Orthop 2(1):1–8. 10.1186/s40634-015-0022-4 10.1186/s40634-015-0022-4 PMC4545757 26914874

[5] Palmer A, Fernquest S, Gimpel M, Birchall R, Judge A, Broomfield J, Newton J, Wotherspoon M, Carr A, Glyn-Jones S (2017). Physical activity during adolescence and the development of cam morphology: a cross-sectional cohort study of 210 individuals. Br J Sports Med.

[6] Nawabi DH, Bedi A, Tibor LM, Magennis E, Kelly BT (2014). The demographic characteristics of high-level and recreational athletes undergoing hip arthroscopy for femoroacetabular impingement: a sports-specific analysis. Arthroscopy.

[7] Agricola R, Heijboer MP, Bierma-Zeinstra SM, Verhaar JA, Weinans H, Waarsing JH (2013). Cam impingement causes osteoarthritis of the hip: a nationwide prospective cohort study (CHECK). Ann Rheum Dis.

[8] Beck M, Kalhor M, Leunig M, Ganz R (2005). Hip morphology influences the pattern of damage to the acetabular cartilage femoroacetabular impingement as a cause of early osteoarthritis of the hip. J Bone Joint Surg Br.

[9] Alradwan H, Philippon MJ, Farrokhyar F, Chu R, Whelan D, Bhandari M, Ayeni OR (2012). Return to preinjury activity levels after surgical management of femoroacetabular impingement in athletes. Arthroscopy.

[10] Griffin DR, Dickenson EJ, Wall PD, Achana F, Donovan JL, Griffin J, Hobson R, Hutchinson CE, Jepson M, Parsons NR (2018). Hip arthroscopy versus best conservative care for the treatment of femoroacetabular impingement syndrome (UK FASHIoN): a multicentre randomised controlled trial. Lancet.

[11] Sansone M, Ahldén M, Jónasson P, Thomeé C, Swärd L, Öhlin A, Baranto A, Karlsson J, Thomee R (2017). Outcome after hip arthroscopy for femoroacetabular impingement in 289 patients with minimum 2-year follow-up. Scand J Med Sci Sports.

[12] Gupta A, Redmond JM, Stake CE, Dunne KF, Domb BG (2016). Does primary hip arthroscopy result in improved clinical outcomes? 2-year clinical follow-up on a mixed group of 738 consecutive primary hip arthroscopies performed at a high-volume referral center. Am J Sports Med.

[13] Öhlin A, Ahldén M, Lindman I, Jónasson P, Desai N, Baranto A, Ayeni OR, Sansone M (2019) Good 5-year outcomes after arthroscopic treatment for femoroacetabular impingement syndrome. Knee Surg sports Traumatol Arthrosc:1-6. 10.1007/s00167-019-05429-y 10.1007/s00167-019-05429-y PMC7148278 30972465

[14] Ardern CL, Glasgow P, Schneiders A, Witvrouw E, Clarsen B, Cools A, Gojanovic B, Griffin S, Khan KM, Moksnes H (2016). 2016 consensus statement on return to sport from the first world congress in sports physical therapy, Bern. Br J Sports Med.

[15] Casartelli NC, Leunig M, Maffiuletti NA, Bizzini M (2015). Return to sport after hip surgery for femoroacetabular impingement: a systematic review. Br J Sports Med.

[16] Reiman MP, Peters S, Sylvain J, Hagymasi S, Mather RC, Goode AP (2018). Femoroacetabular impingement surgery allows 74% of athletes to return to the same competitive level of sports participation but their level of performance remains unreported: a systematic review with meta-analysis. Br J Sports Med.

[17] Christian RA, Lubbe RJ, Chun DS, Selley RS, Terry MA, Hsu WK (2019). Prognosis following hip arthroscopy varies in professional athletes based on sport. Arthroscopy.

[18] Frangiamore SJ, Mannava S, Briggs KK, McNamara S, Philippon MJ (2018). Career length and performance among professional baseball players returning to play after hip arthroscopy. Am J Sports Med.

[19] Menge TJ, Bhatia S, McNamara SC, Briggs KK, Philippon MJ (2017). Femoroacetabular impingement in professional football players: return to play and predictors of career length after hip arthroscopy. Am J Sports Med.

[20] Naal FD, Miozzari HH, Kelly BT, Magennis EM, Leunig M, Noetzli HP (2013). The hip sports activity scale (HSAS) for patients with femoroacetabular impingement. Hip Int.

[21] Sansone M, Ahldén M, Jonasson P, Thomeé C, Swärd L, Baranto A, Karlsson J, Thomeé R (2014). A Swedish hip arthroscopy registry: demographics and development. Knee Surg Sports Traumatol Arthrosc.

[22] Griffin DR, Parsons N, Mohtadi NG, Safran MR, Network MAotHOR (2012). A short version of the international hip outcome tool (iHOT-12) for use in routine clinical practice. Arthroscopy.

[23] Thorborg K, Hölmich P, Christensen R, Petersen J, Roos EM (2011). The Copenhagen hip and groin outcome score (HAGOS): development and validation according to the COSMIN checklist. Br J Sports Med.

[24] Jónasson P, Baranto A, Karlsson J, Swärd L, Sansone M, Thomeé C, Ahldén M, Thomeé R (2014). A standardised outcome measure of pain, symptoms and physical function in patients with hip and groin disability due to femoro-acetabular impingement: cross-cultural adaptation and validation of the international hip outcome tool (iHOT12) in Swedish. Knee Surg Sports Traumatol Arthrosc.

[25] Thomeé R, Jónasson P, Thorborg K, Sansone M, Ahldén M, Thomeé C, Karlsson J, Baranto A (2014). Cross-cultural adaptation to Swedish and validation of the Copenhagen hip and groin outcome score (HAGOS) for pain, symptoms and physical function in patients with hip and groin disability due to femoro-acetabular impingement. Knee Surg Sports Traumatol Arthrosc.

[26] Öhlin A, Jónasson P, Ahldén M, Thomeé R, Baranto A, Karlsson J, Sansone M (2019). The hip sports activity scale for patients with femoroacetabular impingement syndrome—validation in Swedish. Transl Sports Med.

[27] Nwachukwu BU, Chang B, Beck EC, Neal WH, Movassaghi K, Ranawat AS, Nho SJ (2019). How should we define clinically significant outcome improvement on the iHOT-12?. HSS J.

[28] Ishøi L, Thorborg K, Kraemer O, Hölmich P (2018). Return to sport and performance after hip arthroscopy for femoroacetabular impingement in 18-to 30-year-old athletes: a cross-sectional cohort study of 189 athletes. Am J Sports Med.

[29] Tijssen M, van Cingel R, de Visser E, Nijhuis-van der Sanden M (2016). A clinical observational study on patient-reported outcomes, hip functional performance and return to sports activities in hip arthroscopy patients. Phys Ther Sport.

[30] Worner T, Thorborg K, Stalman A, Webster KE, Momatz Olsson H, Eek F (2018). High or low return to sport rates following hip arthroscopy is a matter of definition?. Br J Sports Med.

[31] Kraft CN, Pennekamp PH, Becker U, Young M, Diedrich O, Lüring C, von Falkenhausen M (2009). Magnetic resonance imaging findings of the lumbar spine in elite horseback riders: correlations with back pain, body mass index, trunk/leg-length coefficient, and riding discipline. Am J Sports Med.

[32] Svenska Ridsport Förbundet (2019) Landslag hoppning 2019. http://ridsport.se/Grenar/Hoppning/Landslag/ [Accessed 9 dec 2019]

[33] Lund B, Mygind-Klavsen B, Grønbech Nielsen T, Maagaard N, Kraemer O, Hölmich P, Winge S, Lind M (2017). Danish hip arthroscopy registry (DHAR): the outcome of patients with femoroacetabular impingement (FAI). J Hip Preserv Surg.

[34] Menge TJ, Briggs KK, Philippon MJ (2016). Predictors of length of career after hip arthroscopy for Femoroacetabular impingement in professional hockey players. Am J Sports Med.

[35] Sansone M, Ahldén M, Jonasson P, Thomeé C, Swärd L, Baranto A, Karlsson J, Thomeé R (2015). Good results after hip arthroscopy for femoroacetabular impingement in top-level athletes. Orthop J Sports Med.

[36] Shibata KR, Matsuda S, Safran MR (2017). Arthroscopic hip surgery in the elite athlete: comparison of female and male competitive athletes. Am J Sports Med.

[37] Lindman I, Öhlin A, Desai N, Samuelsson K, Ayeni OR, Hamrin Senorski E, Sansone M (2020). Five-year outcomes after arthroscopic surgery for Femoroacetabular impingement syndrome in elite athletes. Am J Sports Med.

