# Supplementary material for: Low rate of high-level athletes maintained a return to pre-injury sports two years after arthroscopic treatment for femoroacetabular impingement syndrome
Source: J Exp Orthop. 2020 Jun 25;7:44. doi: 10.1186/s40634-020-00263-5 (PMC7316920; doi:10.1186/s40634-020-00263-5)
Supplement: Supplementary file 1 — Additional file 1. [file 40634_2020_263_MOESM1_ESM.pdf]

Table A1. Hip Sports Activity Scale (HSAS) Activity Score, English Version[a]

8	**Competitive sports (elite level):** Soccer, ice hockey, field hockey, American football/rugby, martial arts, tennis, track and field, indoor sports[b], beach volleyball, lacrosse, baseball/softball
7	**Competitive sports (elite level):** Downhill skiing, snowboarding<br>**Competitive sports (minor leagues/collegiate**): Soccer, ice hockey, field hockey, American football/rugby, martial arts, tennis, track and field, indoor sports[b], beach volleyball, lacrosse, baseball/softball
6	**Competitive sports (elite level):** Golf, bicycle racing, mountain biking, swimming, rowing, cross-country skiing/biathlon, horseback riding, cricket<br>**Competitive sports (minor leagues/collegiate):** Downhill skiing, snowboarding
5	**Competitive sports (minor leagues/collegiate):** Golf, bicycle racing, mountain biking, swimming, rowing, cross- country skiing/biathlon, horseback riding, cricket
4	Recreational sports: Tennis, downhill skiing, snowboarding, indoor sports[b], baseball/softball
3	Recreational sports: Aerobics, jogging, lower extremity weightlifting, horseback riding, cricket
2	Recreational sports: Golf, bicycle riding, mountain biking, swimming, rowing, cross-country skiing/biathlon, dancing, inline skating
1	Recreational sports: Swimming, cycling, hiking, Nordic walking (quick walking with ski poles)
0	No recreational or competitive sports
	Please indicate your preferred sport:

[a] From Naal et al. (2013)
[b] Indoor sports: basketball, squash, racquetball, handball, badminton, volleyball
